# Unveiling risk factors: a prognostic model of frequent peritonitis in peritoneal dialysis patients

**DOI:** 10.3389/fmed.2025.1456857

**Published:** 2025-01-29

**Authors:** Qi-jiang Xu, Zhi-yun Zang, Xue-li Zhou, Ni-ya Ma, Li Pu, Zi Li

**Affiliations:** ^1^Department of Nephrology, Institute of Nephrology, West China Hospital of Sichuan University, Chengdu, China; ^2^Department of Nephrology, Yibin Second People’s Hospital, Yibin, China; ^3^Nephrology Department of West China Hospital, School of Nursing, Sichuan University, Chengdu, China

**Keywords:** peritoneal dialysis, peritonitis, frequent episodes, prediction model, nomogram

## Abstract

**Introduction:**

Peritoneal dialysis-associated peritonitis (PDAP) is a serious complication of peritoneal dialysis (PD) patients. The aim of this study was to construct a risk prediction model for frequent episodes in PDAP patients.

**Methods:**

This retrospective cohort study included PDAP patients in our center from January 1, 2010 to December 31, 2021. The risk prediction model for frequent episodes in PDAP patients was constructed by the binary logistic regression.

**Results:**

We included 371 PDAP patients, of which 235 patients had single episode and 136 had frequent episodes. We randomly allocated the patients into training set (296 patients) and test set (75 patients) in the ratio of 8:2. In the training set, we found several independent risk factors significantly associated with frequent episodes in PDAP patients, including diabetes mellitus (DM), hemoglobin (HB), serum albumin (ALB), lactatic dehydrogenase (LDH), serum potassium (K), N-terminal pro-brain natriuretic peptide (NT-proBNP) and peritoneal dialysate white cell counts on day 1. And we constructed a prediction model with an area under curve (AUC) values of 0.75 in the training set and 0.76 in the test set, which showed excellent predictive performance.

**Conclusion:**

We constructed a predictive model that demonstrated excellent predictive performance for identifying high-risk frequent episodes in PDAP patients and developed a more intuitive nomogram for evaluating the risk. However, multicenter studies with a larger sample size are warranted to validate the model in the future.

## Introduction

Peritoneal Dialysis (PD) is an effective renal replacement therapy for patients with end-stage renal failure (1, 2). However, its serious and common complication, peritoneal dialysis associated peritonitis (PDAP), can significantly impact the prognosis of PD patients (3–5). Studies have shown that PDAP is an independent predictor of failure of peritoneal dialysis technique, resulting in 20.7–21.4% patients having catheter removal, switch to hemodialysis or death (6, 7).

Several studies reported that a considerable number of patients (20.5–31.0%) would have recurrent or relapse peritonitis after the first peritonitis, and the patients had poorer prognosis and longer treatment period when comparing to the control group (first peritonitis episode without relapse or recurrence) (8–11). Relapsed peritonitis or recurrent peritonitis was associated independently with catheter removal and permanent transfer to hemodialysis therapy (9). Further researchers found that higher risk of subsequent peritonitis was related to individual characteristics [obesity (12), black race (13), diabetes mellitus (DM) (13)] and biofilm produced by bacteria (14). Therefore, identifying the high-risk population of frequent episodes has important clinical value for improving the management and prognosis of PDAP patients.

In recent years, prediction models based on a large number of clinical datasets and multiple indicators can help clinicians to predict the prognosis of diseases. Our recent retrospective study constructed risk prediction models by multiple machine learning algorithms to predict technique failure in PDAP patients (15). A multicenter and retrospective cohort study conducted in Thailand has developed a risk prediction tool for peritonitis-associated technique failure based on multivariate logistic regression (16). Meng et al. established a nomogram for predicting peritonitis cure in PDAP patients using multivariate logistic regression (17). However, there’s no any prediction model to assess the risk of frequent peritonitis in PDAP patients.

The objective of the present study was to investigate the risk factors for frequent peritonitis and construct a prediction model, which may help identify high-risk patients and adjust treatment regimens timely.

## Methods

### Study population

We conducted a retrospective study of patients who were diagnosed with PDAP and admitted to West China Hospital of Sichuan University from January 1, 2010 to December 31, 2021. The diagnosis of PDAP was defined according to the 2022 International Society for Peritoneal Dialysis (ISPD) guidelines (18). Exclusion criteria included (1) age < 18 years old, (2) withdraw from PD within 2 years after the first PDAP due to catheter removal, transfer to hemodialysis, kidney transplantation, or death, (3) patients who failed to follow-up, (4) patients without clinical data about peritoneal dialysates (absence of the peritoneal dialysate white cell count or the results of pathogenic bacteria culture).

The single group was defined as only one episode of PDAP during the 2-year follow-up period. The frequent group was defined as the episode of PDAP with 2 or more times during the period. Subsequently, PDAP patients were categorized into the single group or frequent group based on their episode frequency.

This study was approved by the Medical Ethics Committee of West China Hospital of Sichuan University (No. 2019-33), and was registered at the Thai Clinical Trials Registry (TCTR20180313004). This study followed the Declaration of Helsinki, and written informed consents were obtained from all participants.

### Data collection

From the medical records and laboratory information system, we collected the following clinical and laboratory data. The demographic features included age, gender, height, weight, systolic blood pressure (SBP), diastolic blood pressure (DBP), dialysis vintage, hospital stay, fever, and comorbidities, which included DM, cirrhosis, cardiovascular diseases (CVDs), connective tissue diseases, etc. And the body mass index (BMI) was calculated based on height and weight. The Charlson Comorbidity Index Score (CCI) was calculated based on the patients’ age and comorbidities (19). The laboratory data included hemoglobin (HB), white blood cell (WBC), serum albumin (ALB), serum creatinine (SCr), blood urea nitrogen (BUN), uric acid (UA), estimated glomerular filtration rate (eGFR), lactate dehydrogenase (LDH), aspartate aminotransferase (AST), glutamyl transpeptidase (GGT), hydroxybutyrate dehydrogenase (LBDH), β2-microglobulin (β2-MG), serum potassium (K), N-terminal pro-brain natriuretic peptide (NT-proBNP), high-sensitive C-reactive protein (hs-CRP), interleukin 6 (IL-6), procalcitonin (PCT), etc. The peritoneal dialysate white cell counts on day 1, day 3 and day 5 were collected. Furthermore, the results of causative organisms, the regimens of intraperitoneal (IP) and intravenous antibiotic were recorded. The classifications of causative organisms and IP antibiotic regimens were based on the 2022 ISPD guidelines (18).

### Statistical analysis

The statistical analysis was performed by the IBM SPSS Statistics version 25.0 (IBM Corp., Armonk, NY, United States). After the analysis of data normality by the Kolmogorov–Smirnov test, the normal distribution data were expressed by mean ± standard deviation (SD) and the non-normal distribution data were represented by median and interquartile range (IQR). And the count data was expressed as the number of cases (%). Categorical variables were analyzed by Chi-square test or Fisher’s exact test, while continuous variables were analyzed by Student’s *t* test or Mann–Whitney U test. A two-sided *p* value less than 0.05 was considered statistically significant. Of all patients, 80% of them were randomly allocated to the training set and constructed a prediction model, while the remaining 20% were assigned to the test set for internal validation. In the training cohort, univariate analysis was conducted to select the risk factors of frequent PDAP. Variables with *p* < 0.05 in the univariate analysis were included in the multivariate analysis. And the binary logistic regression analysis was used in the construction of prediction model, and a nomogram was established which was possible to assess the risk of frequent episodes in PDAP patients intuitively. And the receiver operating characteristic curves (ROC) and area under curve (AUC) were presented for the assessment of prediction model’s accuracy and clinical utility.

## Results

**Figure 1:**
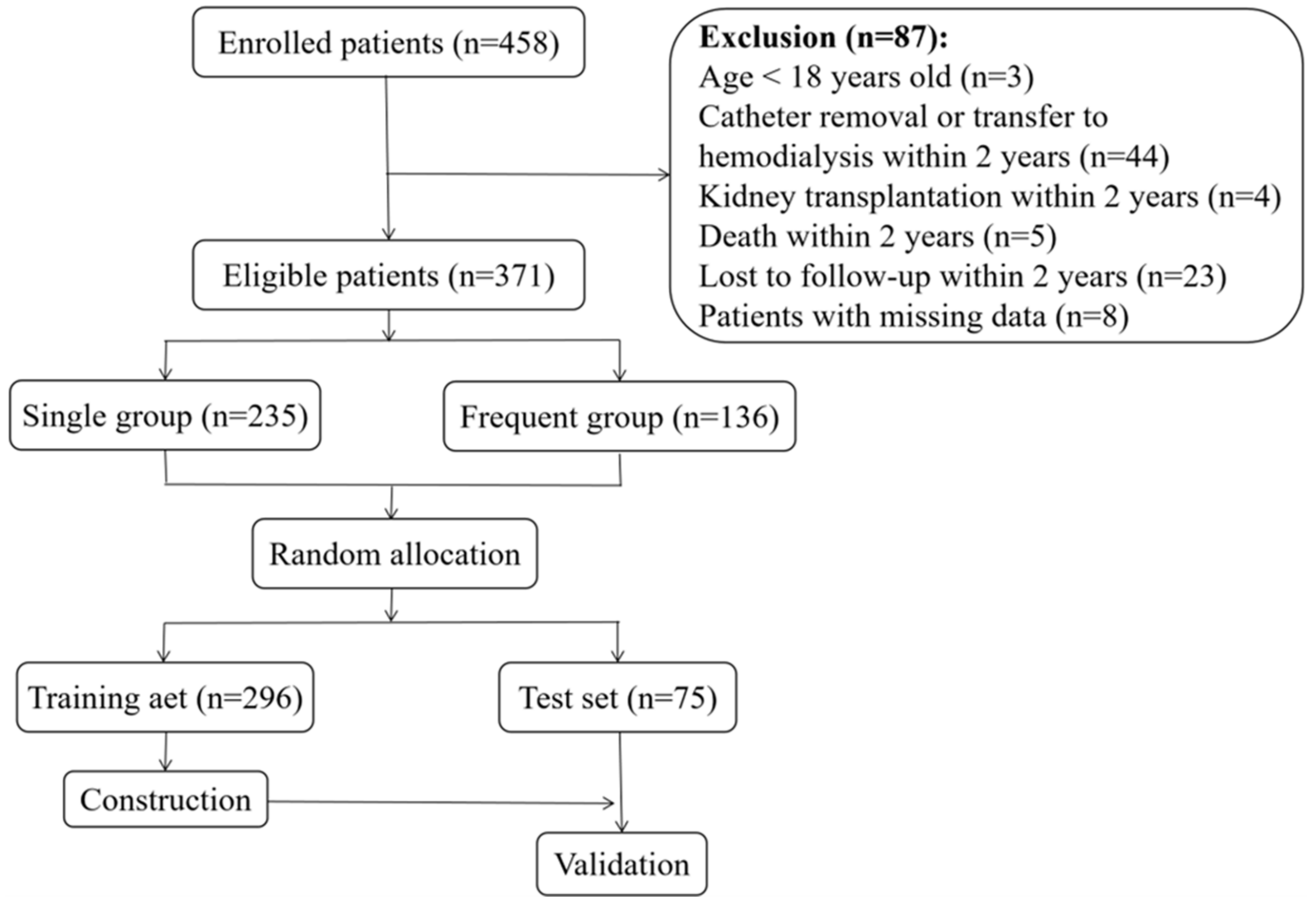
Flow chart.

The flow chart of this retrospective study is presented in Figure 1. A total of 458 PDAP patients were enrolled in our study. However, 87 patients were excluded: 3 patients were younger than 18 years old, 2 patients missing the results of causative organisms, 6 patients missing the peritoneal dialysate white cell count, and 23 patients lost to follow-up. And there were 53 patients quit from PD after their first PDAP due to catheter removal, transfer to hemodialysis, kidney transplantation, or death.

### Baseline characteristics

**Figure 2:**
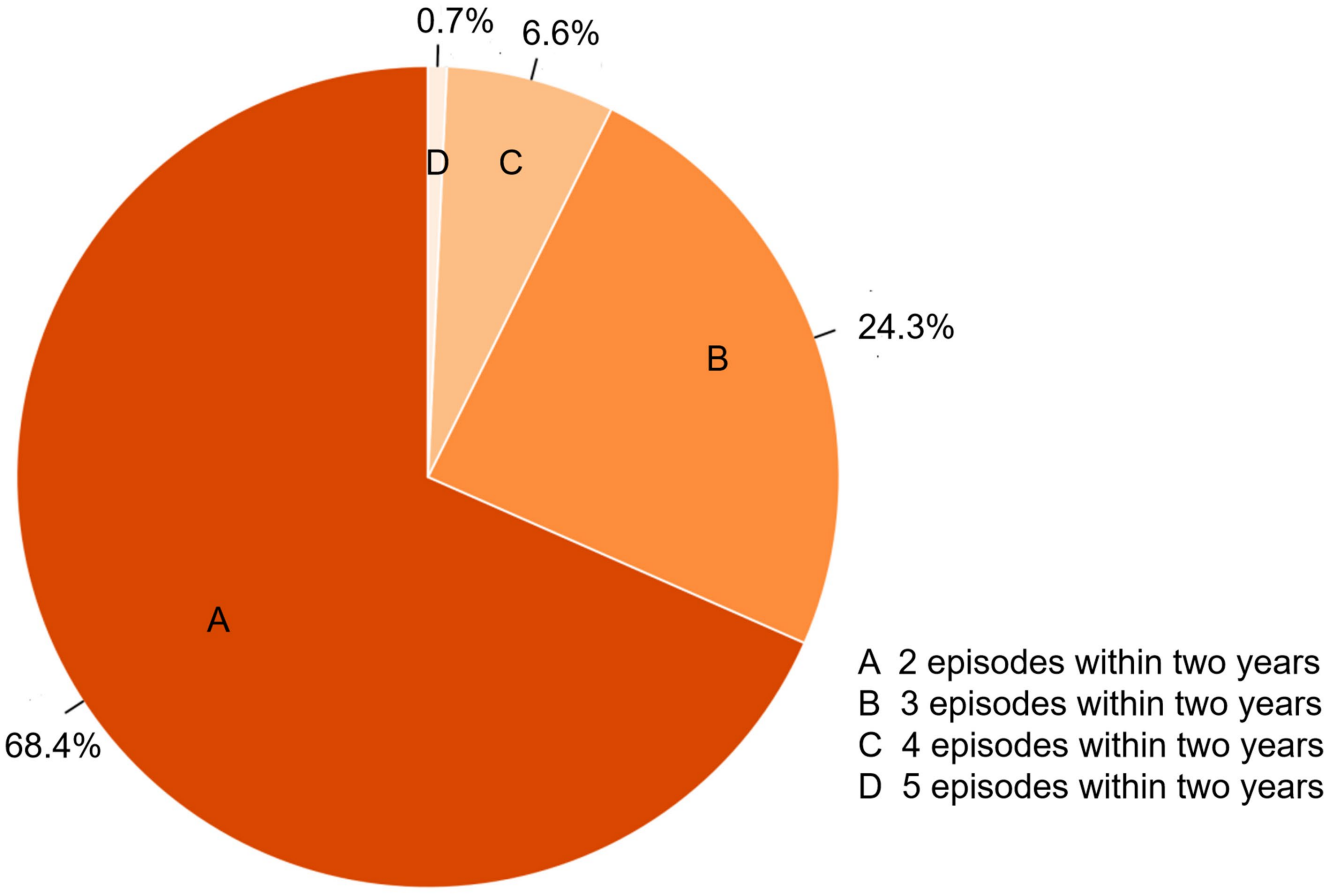
The frequency of PDAP patients in the frequent group.

After rigorous screening, a total of 371 eligible patients were included in the study, with 235 patients in the single group and 136 patients in the frequent group. Figure 2 shows the frequency of PDAP patients in the frequent group, varying from 2 episodes to 5 episodes within 2 years. For the majority of these patients, 93 patients had 2 episodes within 2 years (68.4%), 33 patients having 3 episodes within 2 years (24.3%), 9 patients having 4 episodes within 2 years (6.6%), and one patients having 5 episodes within 2 years (0.7%).

The participants were divided into the training set of 296 (194 single group, 102 frequent group) and the test set of 75 (41 single group, 34 frequent group). In the training set, the median age was 48.94 years old and there were 151 male patients which accounted for 51.0%. The median PD duration was 485.5 days. Among the eligible patients, 74 patients (25.0%) had DM, and the median score of CCI was 3 points (Table 1). In the training cohort, the comparison between the two groups showed significant differences in CCI score, BMI, and DM (*p* < 0.01) (Table 1). Hematological analysis (Table 2) showed a significant difference in HB (*p* = 0.005). Biochemical variables (Table 2) such as AST, ALB, GGT, LDH, HBDH, K, transferrin, β2-MG, and NT-proBNP showed significant differences (*p* < 0.05).

**Table 1:** Baseline demographic characteristics in the training set.

Variables	Total (*n* = 296)	Single group (*n* = 194)	Frequent group (*n* = 102)	*p* value*
Age, year, ± SD	48.94 ± 14.60	48.14 ± 14.52	50.45 ± 14.64	0.195
CCI score, points, ± SD	3 ± 1	3 ± 1	4 ± 1	**0.000**
Male, *n*%	151 (51.0)	102 (52.6)	49 (48.0)	0.458
BMI, kg/m^2^, ± SD	22.63 ± 3.30	22.02 ± 2.98	23.77 ± 3.58	**0.000**
Dialysis vintage, days, IQR	485.5 (191.8, 1168.0)	455.0 (206.8, 1149.0)	521.5 (154.5, 1237.8)	0.885
Fever, *n*%	21 (7.1)	12 (6.2)	9 (0.9)	0.401
SBP, mmHg, ± SD	173.77 ± 24.58	136.40 ± 23.77	140.37 ± 25.97	0.187
DBP, mmHg, ± SD	85.96 ± 16.40	85.86 ± 15.90	86.16 ± 17.38	0.881
Hospital stay, days, ± SD	15.02 ± 7.23	15.01 ± 7.28	15.04 ± 7.17	0.974
DM, *n*%	74 (25.0)	26 (13.4)	48 (47.1)	**0.000**

*Comparison between single group and frequent group.

Bold values, *p* < 0.05.

**Table 2:** Baseline characteristics of laboratory variables in the training set.

Variables	Total (*n* = 296)	Single group (*n* = 194)	Frequent group (*n* = 102)	*p* value*
HB, g/L, ± SD	97.56 ± 20.69	99.97 ± 21.27	92.93 ± 18.77	**0.005**
PLT, 10^9^/L, ± SD	202.67 ± 92.18	203.80 ± 90.66	200.52 ± 95.41	0.771
WBC, 10^9^/L, ± SD	7.81 ± 3.77	7.77 ± 3.91	7.90 ± 3.50	0.765
DB, μmol/L, IQR	1.80 (1.20, 2.50)	1.80 (1.28, 2.50)	1.80 (1.20, 2.40)	0.940
IB, μmol/L, ± SD	3.66 ± 2.33	3.65 ± 2.38	3.69 ± 2.25	0.904
ALT, IU/L, IQR	11.00 (8.00, 18.00)	12.00 (8.00, 17.00)	11.00 (8.00, 21.00)	0.789
AST, IU/L, ± SD	19.52 ± 10.20	18.39 ± 7.99	21.69 ± 13.26	**0.023**
ALB, g/L, ± SD	31.39 ± 4.63	32.21 ± 4.33	29.82 ± 4.80	**0.000**
GLB, g/L, ± SD	27.32 ± 5.53	27.49 ± 5.72	26.99 ± 5.15	0.462
GLU, mmol/L, ± SD	6.22 ± 2.92	6.38 ± 3.17	5.91 ± 2.35	0.186
BUN, mmol/L, IQR	17.70 (14.30, 22.00)	17.30 (13.92, 21.35)	18.49 (15.27, 23.30)	0.113
SCr, μmol/L, ± SD	854.37 ± 294.52	854.50 ± 280.14	854.11 ± 321.56	0.991
Cys-C, mg/L, IQR	5.76 (4.64, 6.80)	5.68 (4.65, 6.82)	5.81 (4.64, 6.80)	0.909
eGFR, ml/ (min*1.73m^2^), ± SD	5.86 ± 2.44	5.86 ± 2.36	5.85 ± 2.68	0.973
UA, μmol/L, ± SD	349.98 ± 97.34	345.31 ± 96.51	359.04 ± 98.77	0.253
TG, mmol/L, IQR	1.28 (0.93, 1.85)	1.29 (0.96, 1.72)	1.22 (0.90, 1.97)	0.902
CHOL, mmol/L, IQR	4.04 (3.37, 4.78)	4.00 (3.35, 4.78)	4.09 (3.47, 4.76)	0.730
HDL-C, mmol/L, ± SD	1.26 ± 0.67	1.23 ± 0.71	1.34 ± 0.59	0.156
LDL-C, mmol/L, IQR	2.16 (1.75, 2.80)	2.20 (1.78, 2.79)	2.09 (1.69, 2.86)	0.583
ALP, IU/L, ± SD	79.73 ± 47.20	80.59 ± 43.14	78.10 ± 54.05	0.669
GGT, IU/L, IQR	22.00 (15.00, 36.00)	20.00 (13.75, 34.00)	25.00 (17.00, 39.00)	**0.023**
CK, IU/L, IQR	79.00 (48.00, 148.50)	78.00 (46.25, 157.50)	80.00 (50.50, 135.00)	0.870
LDH, IU/L, ± SD	205.21 ± 48.79	189.54 ± 53.53	235.00 ± 73.80	**0.000**
HBDH, IU/L, ± SD	160.42 ± 55.94	153.19 ± 51.48	175.68 ± 61.92	**0.003**
Na, mmol/L, ± SD	139.07 ± 4.23	138.94 ± 4.26	139.30 ± 4.20	0.499
K, mmol/L, ± SD	3.86 ± 0.69	3.98 ± 0.73	3.64 ± 0.55	**0.000**
Cl, mmol/L, ± SD	96.76 ± 5.60	96.91 ± 5.68	96.49 ± 5.44	0.536
Carbon dioxide binding force, mmol/L, ± SD	26.04 ± 3.38	25.98 ± 3.44	26.13 ± 3.28	0.727
Ca, mmol/L, ± SD	2.16 ± 0.23	2.16 ± 0.19	2.17 ± 0.28	0.618
Mg, mmol/L, ± SD	0.86 ± 0.20	0.86 ± 0.20	0.86 ± 0.21	0.755
P, mmol/L, ± SD	1.45 ± 0.57	1.42 ± 0.48	1.51 ± 0.70	0.225
Transferrin, g/L, ± SD	1.63 ± 0.75	1.53 ± 0.40	1.81 ± 1.11	**0.030**
Prealbumin, mg/L, ± SD	311.59 ± 107.48	294.97 ± 83.46	330.34 ± 127.93	0.146
β2-MG, mg/dL, ± SD	28.03 ± 9.14	26.03 ± 7.72	30.72 ± 10.32	**0.046**
hs-CRP, mg/L, IQR	23.15 (4.12, 86.58)	28.50 (4.40, 94.75)	15.60 (2.76, 67.25)	0.262
IL-6, pg./mL, IQR	15.24 (7.76, 53.63)	19.94 (8.36, 60.08)	11.90 (6.49, 29.84)	0.102
NT-proBNP, pg./mL, IQR	7,406 (2,779, 22,457)	4,486 (2,153, 12,770)	13,156 (6,156, 32,118)	**0.019**
PCT, ng/mL, IQR	1.98 (0.54, 7.80)	2.08 (0.57, 8.62)	1.30 (0.43, 7.80)	0.347
iPTH, pmol/L, IQR	19.68 (8.12, 36.94)	18.62 (7.86, 37.34)	19.82 (9.51, 36.72)	0.969

*Comparison between single group and frequent group.

Bold values, *p* < 0.05.

In addition, we observed a statistically significant difference (*p* < 0.001) in the results of causative organisms between the single and frequent groups, indicating notable variations in microorganism spectrum (Table 3). In the single group, 109 patients yielded negative culture results, whereas 55 patients showed negative outcomes in the frequent group. We found significant statistical differences in polymicrobial peritonitis between the single and frequent groups (*p* < 0.001) by further analysis.

**Table 3:** Causative organisms of peritoneal dialysates in the training set.

Causative organisms	Total (*n* = 296)	Single group (*n* = 194)	Frequent group (*n* = 102)	*p* value*
Culture negative peritonitis, *n*%	164 (55.4)	109 (56.2)	55 (53.9)	0.714
*Staphylococcus aureus*, n%	9 (3.0)	6 (3.1)	3 (2.9)	1.000
Coagulase-negative Staphylococci, *n*%	48 (16.2)	34 (17.5)	14 (13.7)	0.507
Streptococcus, *n*%	19 (6.4)	16 (8.2)	3 (2.9)	0.086
Enterococcus, *n*%	7 (2.4)	5 (2.6)	2 (2.0)	1.000
Corynebacterium, *n*%	5 (1.7)	4 (2.1)	1 (1.0)	0.663
Pseudomonas, *n*%	3 (1.0)	2 (1.0)	1 (1.0)	1.000
Acinetobacter, *n*%	7 (2.4)	4 (2.1)	3 (2.9)	0.696
Enterogenous Gram-negative bacteria, *n*%	18 (6.1)	12 (6.2)	6 (5.9)	1.000
Polymicrobial infection, *n*%	14 (4.7)	0 (0)	14 (13.8)	**0.000**
Fungus, *n*%	2 (0.7)	2 (1.0)	0 (0)	0.547

*Comparison between single group and frequent group.

Bold values, *p* < 0.05.

The peritoneal dialysate white cell counts in the frequent group on day 1 (3,600 × 10^6^/L vs. 1,410 × 10^6^/L, *p* < 0.001) and on day 5 (55 × 10^6^/L vs. 40 × 10^6^/L, *p* = 0.021) were significantly higher than the single group (Table 4). Table 5 shows the antibiotic regimens during hospitalization. We found that there was no significant difference in the initial IP antibiotic regimens between the single and frequent groups (*p* = 0.059). What’s more, our results demonstrated that patients in the frequent group used more types of antibiotics (*p* < 0.001), and upgraded antibiotics (*p* = 0.009), using intravenous antibiotics (*p* < 0.001) and glycopeptides (*p* = 0.029) more frequently.

**Table 4:** Baseline characteristics of peritoneal dialysate white cell counts in the training set.

Variables	Total (*n* = 296)	Single group (*n* = 194)	Frequent group (*n* = 102)	*p* value*
Day 1, 10^6^/L, IQR	2,420 (702, 5,870)	1,410 (232, 4,124)	3,600 (1,114, 7,950)	**0.000**
Day 3, 10^6^/L, IQR	130 (28, 260)	130 (30, 483)	130 (24, 49)	0.948
Day 5, 10^6^/L, IQR	47 (20, 163)	40 (10, 150)	55 (30, 188)	**0.021**

*Comparison between single group and frequent group.

Bold values, *p* < 0.05.

**Table 5:** The antibiotic regimens during hospitalization in the training set.

Variables	Total (*n* = 296)	Single group (*n* = 194)	Frequent group (*n* = 102)	*p* value*
Initial IP antibiotic regimens				0.059
1st-generation cephalosporin and 3rd-generation cephalosporin, *n*%	234 (79.0)	162 (83.5)	72 (70.6)	
1st-generation cephalosporin and aminoglycoside, *n*%	18 (6.1)	8 (4.1)	10 (9.8)	
1st-generation cephalosporin and other Gram-negative antibiotic, *n*%	8 (2.7)	2 (1.0)	6 (5.9)	
Vancomycin and 3rd-generation cephalosporin, n%	15 (5.1)	8 (4.1)	7 (6.9)	
Vancomycin and aminoglycoside, *n*%	7 (2.4)	5 (2.6)	2 (2.0)	
Vancomycin and other Gram-negative antibiotic, *n*%	6 (2.0)	3 (1.6)	3 (2.8)	
Other Gram-positive antibiotic and 3rd-generation cephalosporin, *n*%	2 (0.7)	1 (0.5)	1 (1.0)	
Other Gram-positive antibiotic and other Gram-negative antibiotic, *n*%	6 (2.0)	5 (2.6)	1 (1.0)	
Upgraded antibiotics, *n*%	85 (28.7)	46 (23.7)	39 (38.2)	**0.009**
Use of glycopeptides, *n*%	84 (28.4)	47 (24.2)	37 (36.3)	**0.029**
Categories of antibiotics, types, IQR	2 (2, 3)	2 (2, 3)	3 (2, 4)	**0.000**
Intravenous antibiotics, *n*%	99 (33.4)	49 (25.3)	52 (60.0)	**0.000**

Other Gram-negative antibiotics include metronidazole, ciprofloxacin, erythromycin, aztreonam, and co-trimoxazole. Other Gram-positive antibiotics include amoxicillin, ampicillin, teicoplanin, and erythromycin.

*Comparison between single group and frequent group.

Bold values, *p* < 0.05.

### Construction of prediction model

**Figure 3:**
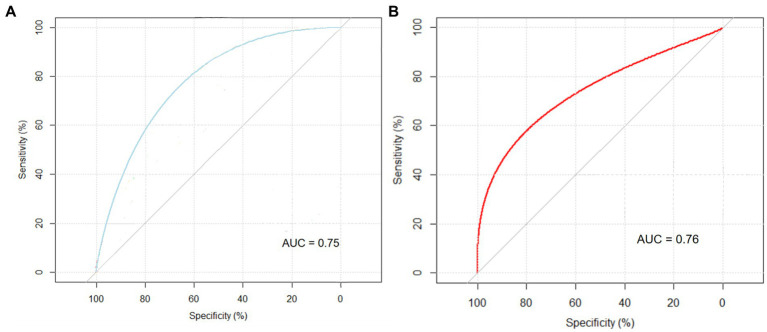
ROC curves of the prediction model. **(A)** Training set. **(B)** Test set.

**Figure 4:**
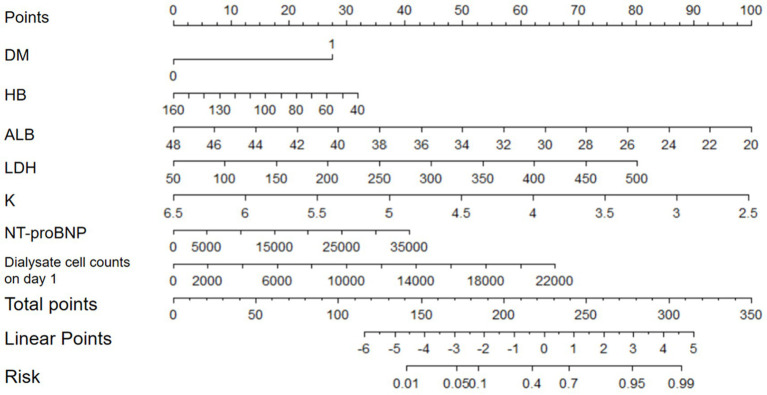
Nomogram for predicting the risk of frequent episodes in PDAP patients.

Variables that showed significant differences in univariate analysis were included in the binary logistic regression model to identify risk factors which was associated with frequent episodes of PDAP. We found several important risk factors which were DM, HB, ALB, LDH, K, NT-proBNP and peritoneal dialysate white cell counts on day 1 after the multivariate analysis, and the regression coefficients (B) corresponding to each risk factor were showed in Table 6. Then, based on the regression coefficients, we constructed a comprehensive predictive index model (L): L = 1.57 × DM − 0.02 × HB − 0.19 × ALB + 0.01 × LDH − 1.36 × K + 0.01 × NT-proBNP + 0.01 × peritoneal dialysate white cell counts on day 1. And this prediction model constructed by the 7 risk factors had the AUC of 0.75 (95% Confidence interval (CI) 0.67–0.84; *p* < 0.001) in the training set (Figure 3A). Furthermore, we constructed a nomogram which could assess the individual risk of frequent episodes in PDAP patients more intuitively (See Figure 4).

**Table 6:** Multivariate analysis of frequent episodes in PDAP patients.

Variables	B	*p* value	OR	95% CI
DM	1.57	0.005	4.80	1.61–14.31
HB	−0.02	0.031	0.98	0.95–0.99
ALB	−0.19	0.004	0.82	0.72–0.94
LDH	0.01	0.032	1.01	1.01–1.02
K	−1.36	0.007	0.26	0.10–0.68
NT-proBNP	0.01	0.021	1.01	1.01–1.02
Peritoneal dialysate white cell counts on day 1	0.01	0.013	1.01	1.01–1.02

### Validation of prediction model

A subset of 75 patients was incorporated into the validation analysis. The AUC value of the prediction model in the test set was 0.76 (95% CI 0.56–0.97, *p* = 0.041) (see Figure 3B). This predictive model demonstrated excellent performance in both the training and test sets.

## Discussion

We constructed a prediction model and a nomogram that assessed the risk of frequent episodes of PDAP by utilizing binary logistic regression. This is the first study to predict the risk of frequent episodes and identify high-risk PDAP patients by developing a prediction model. What’s more, our research has revealed a plethora of valuable independent risk factors associated with frequent episodes in PDAP patients. Based on the nomogram and these risk factors, clinicians can calculate the probability of patients developing frequent episodes easily, which is beneficial for evaluating the prognosis and guiding treatment regimens of PDAP patients.

Previous studies have developed prediction models for PDAP patients using logistic regression (16, 17). There were several studies exploring the risk factors in relapse or recurrent PDAP patients. A retrospective study included 181 participants who experienced 339 episodes revealed that the risk of relapse and recurrent peritonitis in patients with creatinine clearance >5 mL/min was significantly higher when comparing to anuric patients (odds ratio (OR) 6.76; 95% CI, 1.90–23.8) (20). The observational cohort study based on Australian and New Zealand Dialysis and Transplant Registry data found that the category of causative organisms was associated with frequent episodes (9). Nevertheless, there were no prediction models about frequent episodes in PDAP patients.

We constructed the prediction model by binary logistic regression, and the model’s results could be directly represented as probability, which was crucial for understanding and interpreting the predictive results (21, 22). In our study, we constructed a nomogram displaying the probability more intuitively. In addition, the binary logistic regression prediction model does not require the assumption of linear relationships between variables, which exhibits an advantage in handling non-linear datasets in clinic. Furthermore, logistic regression shows robustness because of being less sensitive to outliers (23). Therefore, the binary logistic regression model holds significant advantages in specific environments and conditions.

In our prediction model, we found several important predictors of frequent episodes. DM was a significant risk factor for PDAP patients with frequent episodes. Some studies have revealed DM was linked to an elevated risk of PDAP (13, 24), which is attributed to the immunocompromised state in diabetic patients, rendering them more susceptible to infections (24).

Previous study found that serum albumin was an independent factor associated with peritonitis (*p* = 0.025) (25). PD patients with an initial serum albumin level less than 29 g/L had a peritonitis rate of 1.5 episodes/dialysis-year compared with 0.6 episodes/dialysis-year for patients with ≥29 g/dL (*p* < 0.001) (26). Nevertheless, few studies found the HB was associated with PDAP. One Croatian study suggested that hemoglobin level was correlated with Malnutrition Inflammation Score and serum albumin level in PD patients (27). Comparing to the patients without peritonitis, the peritonitis group had lower hemoglobin (106.7 ± 9.3 g/L vs. 115.1 ± 11.0 g/L, *p* = 0.012) (25). Decreased serum potassium level was a critical indicator of frequent episodes, which was consistent with the previous results (28, 29). And hypokalaemia has been highlighted in the ISPD peritonitis guideline recommendations (18). Therefore, we suggest to reduce the episodes of peritonitis by paying more attention to adjust the anemia, malnutritional and disturbance of electrolyte in PDAP patients.

Our investigations have identified NT-proBNP as a significant determinant of frequent episodes, which was not reported in previous studies about PDAP. During the process of PDAP, the decreased ultrafiltration and subsequent volume overload, could trigger an elevation of NT-proBNP. We advocate continuous monitoring of NT-proBNP throughout the treatment of PDAP and further exploration to unravel the relationship between NT-proBNP and frequent episodes.

Lastly, elevated peritoneal dialysate white cell counts on day 1 increased the likelihood of frequent infections. The previous study has demonstrated that peritoneal dialysate white cell counts on day 3, rather than day 1, could predict treatment failure (30). One study revealed that patients with frequent episodes had significantly higher peritoneal dialysate white cell counts on day 3 and day 5 (31), but the researchers did not collect the data of peritoneal dialysate white cell counts on day 1. In terms of the peritoneal dialysate white cell counts on day 5, we found similar results in the frequent group. Clinicians can assess the risk of frequent episodes of PDAP patients and proactively improve the prognosis of PDAP patients by identifying these important predictors early.

Our study had several limitations. Firstly, as a single-center retrospective study, there is a possibility of selection bias. We did not conduct external validation and the results may not be generalizable to other populations. Secondly, only data during hospitalization was collected and analyzed. Long term follow-up data about frequent episodes of PDAP patients will provide more valuable insights into the reliability and robustness of the model. Thirdly, several novel biomarkers that demonstrated excellent predictive performance for adverse outcomes in PDAP patients could be integrated into future prediction models (32, 33). What’s more, we did not collect the data about exit site infection, which might be a risk factor for frequent episodes.

We constructed a predictive model to assess the risk of frequent PDAP episodes by incorporating seven significant risk factors. And our prediction model showed excellent performance in the training and validation sets. We could identify the high-risk patients with frequent episodes by the available variables in clinic. For the high-risk population, we strongly recommend a comprehensive assessment of relevant predictors, reassessment of antibiotic regimens, and consideration about catheter removal or switch to hemodialysis. However, it must be emphasized that meticulous monitoring is still crucial for low-risk patients.

## Conclusion

Our study developed a prediction model and intuitive nomogram for assessing the risk of frequent PDAP episodes and identifying high-risk patients, which was a valuable tool in optimizing patient management and prognosis. Further refinement and validation of this model in larger prospective cohorts is warranted.

## Data Availability

The raw data supporting the conclusions of this article will be made available by the authors, without undue reservation.
